# Implementing and feasibility testing depression screening using the electronic medical record for patients with type 2 diabetes admitted to the hospital

**DOI:** 10.1002/nop2.181

**Published:** 2018-07-20

**Authors:** Letha M. Joseph, Diane C. Berry, Ann Jessup, Jean Davison, Brian J. Schneider, Jack I. Twersky

**Affiliations:** ^1^ Durham VA Medical Center Durham North Carolina; ^2^ The University of North Carolina at Chapel Hill School of Nursing Chapel Hill North Carolina

**Keywords:** depression, health informatics, medication management, therapy

## Abstract

**Aim:**

Is it feasible to implement a programme to screen for depression in patients admitted to the hospital for diabetes complications and use the electronic medical record to notify providers of their patient's depression score and give suggestions for medication and counselling?

**Design:**

A feasibility study was conducted with patients hospitalized with diabetes and depression in the Durham Veterans Affairs Medical Center, Durham, North Carolina, United States.

**Methods:**

Patients with type 2 diabetes were screened for depression. The healthcare provider was notified via the electronic medical record about the patients' depression scores. The provider discussed options for management of depression with the patient and initiated treatment.

**Results:**

The process of screening for depression at admission, notifying the provider by way of electronic medical record that the patient screened positive for depression with suggestions for medication and counseling was feasible and acceptable to providers and patients.

## 1 INTRODUCTION

Patients with diabetes experience a higher risk of developing depression (Hasan, Manmun, Clavarino, & Kairuz, 2015; Hsu et al., 2012). Depression in adults with diabetes may negatively affect their self‐management and adherence to diabetes treatment, which may lead to poor glycaemic control and microvascular and macrovascular complications (Cummings et al., 2016; Shah, Mezzio, Ho, & Ip, 2015; Singh, Khullar, Singh, Kaur, & Mastana, 2015). Patients with depression report more diabetes symptoms and use healthcare services, such as emergency departments and inpatient services for ambulatory care‐sensitive symptoms, more frequently than patients with diabetes who do not have depression (Davydow et al., 2013). According to a multimorbidity disease cluster cost analysis in the Veterans Health Administration, diabetes with depression accounted for the highest healthcare expenditures compared with other multimorbid conditions (Egede et al., 2015). Thus, it is important to develop an efficient system for patients with diabetes to be screened on admission to the hospital, alert their healthcare providers of their depression scores using the electronic medical record and provide providers with counselling and medication options they can discuss with their patients privately.

## 2 BACKGROUND

The medical literature points to a compelling link between diabetes, depression, and adverse outcomes. Researchers found that depression was associated with a 1.5‐fold increased risk of mortality for patients with diabetes (van Dooren et al., 2013). However, in another study, researchers found that depression in patients with diabetes was often unidentified or inadequately treated (Schierhout et al., 2013). Patients may consider it natural to experience symptoms such as sadness, hopelessness, sleep disturbances, lack of interest, and fatigue while managing a long‐term illness such as diabetes. Patients may not consider these symptoms are related to depression and may not report their symptoms to their providers. Data analysed from the Behavioral Risk Factor Surveillance System reported that nearly 45% of patients with diabetes had undiagnosed depression (Li et al., 2009).

The American Diabetes Association (ADA) supports routine screening for depression as part of standard diabetes management (ADA, 2017). Screening for depression at the time of diagnosis of diabetes, during routine follow‐up visits, on initiation of insulin, during hospitalization, and at the onset of any complications can identify the symptoms of depression (ADA, 2017). Despite evidence‐based recommendations, routine depression screening and management are not always included in the management of patients with diabetes admitted to inpatient medical units. Inpatient providers may be primarily focused on managing the medical illness and may not consider the feasibility of depression screening in acute illness and may defer the screening and follow‐up to outpatient providers. Also, providers may not ask their patients about depression, because of time constraints and a lack of expertise in dealing with psychiatric disorders. Nonetheless, depression in patients with diabetes affects their health‐related quality of life, diabetes self‐management, and healthcare use (ADA, 2017). In health care, there is a significant focus on preventable rehospitalizations. Patients with diabetes and depression tend to have higher healthcare use using the emergency department and inpatient care for concerns that could be addressed in ambulatory care (ADA, 2017).

## 3 DESIGN

The purpose of this study was to explore the feasibility of conducting depression screening and follow‐up for patients with diabetes during their hospitalization and to evaluate the effect of depression management on depression, health‐related quality of life, diabetes self‐management, and all‐cause 30‐day readmission rates of patients with diabetes. We used a one group repeated measures study design. *Management of Depression in Veterans with Diabetes* was a feasibility study conducted between 28 July 2016‐31 December 2016, using the electronic medical record to notify providers of their patient's depression scores and suggest counselling and medication for treatment. Since this was a feasibility study an official power analysis was not conducted.

## 4 METHODS

### 4.1 Setting and participants

The medical‐surgical units of the Durham Veterans Affairs Medical Center (DVAMC) in Durham, North Carolina, United States were the setting for this study. Participants included a convenience sample of veterans aged 18 and above, admitted to the hospitalist medical teams during the study period, with a primary diagnosis of type 2 diabetes and a glycated haemoglobin (HbA1c) of 7.0% or higher. Excluded from the study were patients admitted to other teams, patients who did not have a known diagnosis of type 2 diabetes, or who had type 2 diabetes with HbA1c of less than 7.0%, patients who were already receiving treatment for depression and cognitively impaired patients. Patients with a documentation of dementia or cognitive decline in their problem list in the health records or a documentation supportive of delirium during the hospitalization were considered cognitively impaired and unable to provide informed consent.

### 4.2 Measurement

The instruments used in this study included the Patient Health Questionnaire‐2 (PHQ‐2), the Patient Health Questionnaire‐9 (PHQ‐9) (Kroenke, Spitzer, & Williams, 2001), the Veterans RAND (Research and Development) 12‐item Health Survey (VR‐12) (Kazis et al.., 2006) and the Stanford Diabetes Questionnaire (Stanford University School of Medicine, 2007).

#### 4.2.1 Patient Health Questionnaire

Patients who scored 3 or higher on PHQ‐2 screening were given the PHQ‐9 self‐reported questionnaire consisting of nine questions which inquired about symptoms such as anhedonia, depressed mood, suicidality, and physical symptoms caused by depression (Kroenke et al., 2001). These nine questions assess the criteria used for diagnosis of depression by the *Diagnostic and Statistical Manual of Mental Disorders, Fifth Edition (DSM‐5)*criteria (Trangle et al., 2016).

#### 4.2.2 Veterans RAND (Research and Development) 12‐item Health Survey

The VR‐12 survey questionnaire (Kazis, Selim, Rogers, Qian, & Brazier, 2012) addresses eight domains of physical and mental health including general health perceptions, physical functioning, and activity limitations due to physical and emotional problems, pain, fatigue, social functioning, and mental health. These components are weighed by a computerized scoring program, which provides a physical health summary measure called the Physical Component Summary (PCS) and a mental health summary measure called the Mental Component Summary (MCS). The PCS and MCS scores are standardized using a t‐score transformation. PCS and MCS scores range from 0‐100, where a 0 score indicates the lowest level of health and a score of 100 indicates the highest level of health. A recent Medicare Outcome Study (Selim et al., 2009) reported average PCS and MCS scores of 39.82 (*SD* 12.2) and 50.08 (*SD* 11.4), respectively, which represent the current normal PCS and MCS for clinical applications. A higher PCS and MCS reflects better health‐related quality of life (HRQoL) (Kazis et al., 2012; Selim et al., 2009).

#### 4.2.3 The Stanford Diabetes Questionnaire

The Stanford Diabetes Questionnaire (Stanford University School of Medicine^2007^) consists of diabetes‐specific questions to evaluate patients' health behaviours, health status, healthcare use, and self‐efficacy. The questionnaire was developed by the Stanford University School of Medicine Patient Education Research Center. This self‐reported survey includes questions about patients' perception of general health, physical, and emotional symptoms, influence of physical and emotional health on daily activities, diabetes self‐management activities, such as diet, glucose monitoring, management of low and high blood glucose, medications, follow‐up visits, preventive care, patients' confidence in managing various dimensions of diabetes care, use of emergency department services, and hospitalizations.

#### 4.2.4 Emergency department use and hospitalizations

Patients were asked about their emergency department use and hospitalizations at each encounter. Also, their medical record was reviewed to document emergency department visits and hospitalizations.

### 4.3 Ethics

The Institutional Review Board (IRB) at the Durham Veterans Affairs Medical Center, Durham, North Carolina, United States and the University of North Carolina at Chapel Hill, North Carolina, United States, provided regulatory oversight for the study. Participants signed an informed consent and Health Insurance Portability and Accountability Act (HIPAA) form before enrolling in the study. Procedures were in place to ensure participants' privacy.

### 4.4 Procedures

The researchers met with nurse practitioners, physician assistants, and physicians on the hospitalist team and discussed the increased risk for depression in patients with diabetes and the effect of depression on their health‐related quality of life and diabetes self‐management. The researchers provided a brief overview of the study protocol and distributed laminated pocket cards with sections from the Veterans Affairs/Department of Defense (VA/DoD) Clinical Practice Guidelines on the management of major depressive disorder. These pocket cards included information related to the prescribing and monitoring of common antidepressants.

Potential participants were identified from the electronic health records using the inclusion criteria. The study was discussed in detail in a face‐to‐face, individual encounter and all questions were answered before asking participants to sign the informed consent and HIPPA form. The PHQ‐2 was then administered. Patients who scored ≥3 on the PHQ‐2 were enrolled in the study and they received additional screening using the PHQ‐9, VR‐12, and the Stanford Diabetes Questionnaire.

The results of the screening were discussed with each participant and they were provided with information materials. The patient information materials distributed included ‘What is Major Depression? A VA Fact Sheet providing information on the basic facts, symptoms, treatments, and information for families (Mental Illness Research, 2015) and Depression, which is an easy‐to‐read booklet that explains what depression is, how long it lasts, and how to get help (National Institute of Mental Health, 2013). The provider(s) were then notified about the presence and severity of depression symptoms using the electronic medical record. The provider(s) then discussed depression management options with the participants and created a depression management plan honouring the participant's choice. Depending on the severity of the depression and the participant's acceptance, the provider(s) made referrals to mental health provider(s), prescribed antidepressants, or both. On discharge from the inpatient unit, the participant received a referral to primary care mental health integration services and a scheduled follow‐up appointment in 30 days with their primary care provider, who continued outpatient management for the veteran.

At 8 and 12 weeks following discharge from the hospital, the participant received a telephone call and the PHQ‐9, VR‐12, and the Stanford Diabetes Questionnaire were collected. During the second postdischarge follow‐up, the participants were asked about their experience of depression screening, readiness to receive depression treatment, and details of their adherence to antidepressant medications. Also, data were collected on emergency department usage and hospitalizations.

At the conclusion of the study, an anonymous survey was distributed to the hospitalist providers. The survey consisted of eight open‐ended questions that asked about what they liked or did not like about the study; whether they considered patients with diabetes had an increased risk of developing depression compared with patients who did not have diabetes; whether they believed that screening and management of depression needed to be integrated into the management of patients with diabetes; whether they experienced any difficulty in managing patients with depression; their confidence in managing depression; and any suggestions for screening and managing depression in hospitalized patients with diabetes.

### 4.5 Statistical analysis

Descriptive statistics (means, standard deviations, ranges, and percentages) were used to describe the demographics and the PHQ‐9, VR‐12, and the Stanford Diabetes Questionnaire scores. Paired *t* tests were planned, however, due to the low number of participants these were not reported.

## 5 RESULTS

**Figure 1:**
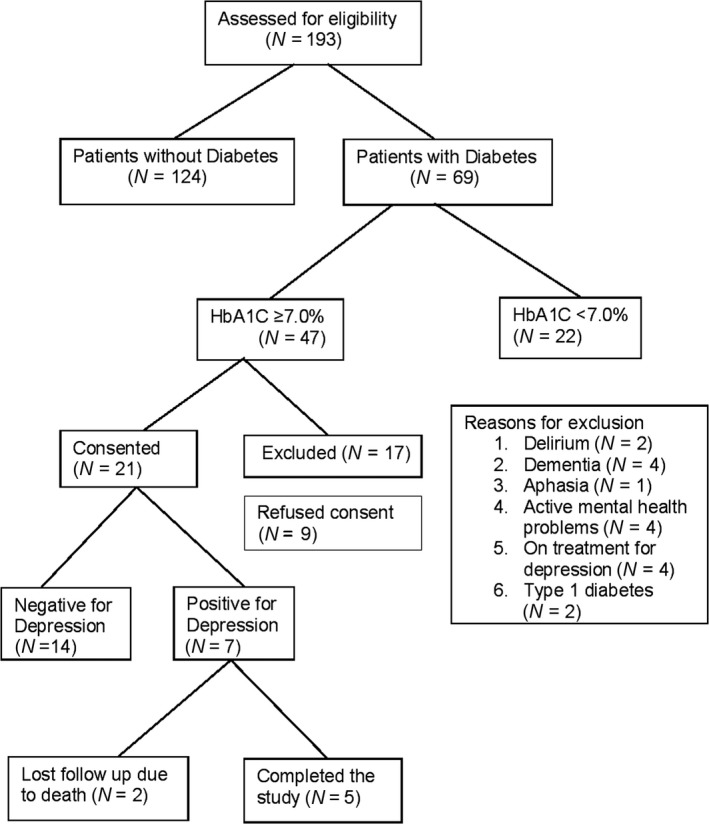
Participant enrollment and completion of the study. Of the 21 patients screened and consented for the study, seven patients qualified and enrolled in the study, and five participants completed the study

Computerized health records of 193 patients admitted to the hospitalist team during the participant enrolment period were screened for eligibility. Of the 193 charts screened, 35.8% of the patients (*N* = 69) had a documented diagnosis of type 2 diabetes in their health records and 60.9% of these patients (*N* = 47) had an HbA1c of 7.0% or higher, thus qualifying them for further review. Seventeen patients were excluded for various reasons (Figure 1). Nine patients declined to enroll in the study. Twenty‐one patients signed the informed consent and answered the PHQ‐2 questionnaire. Seven patients who had PHQ‐2 scores ≥3 qualified for and were enrolled in the study. However, two of the participants were lost to follow‐up due to death. The demographic characteristics of the seven participants who joined the study are in Table 1.

**Table 1:** Demographic characteristics

Total who met inclusion criteria (*N* = 7)		
Age		
Mean	*SD*	Range
65.3	5.7	56–72
Race/Ethnicity		
	Number	Percent
Caucasian	4	57.1
African American	1	14.3
American Indian	1	14.3
Latino	1	14.3
HbA1c		
Mean	*S* *D*	Range
8.8	2.3	7–13.2

HbAlc: Haemoglobin Alc; PHQ‐2: Patient Health Questionnaire‐2 items; *SD*: Standard Deviation.

### 5.1 Patient Health Questionnaire

For the five participants who completed the study, initial PHQ‐9 scores ranged from 12‐17 (mean = 16, *SD* 2.2). Of these five participants, one participant scored in the range of 10–14 (moderate depression) and the remaining four participants scored in the range of 15–19 (moderately severe depression). Four of these participants received antidepressants while inpatient and a prescription to continue the antidepressant after discharge. One participant received an antidepressant and a few sessions of psychotherapy from a clinical psychologist while receiving inpatient rehabilitation.

At the 8 weeks' postdischarge follow‐up, the PHQ‐9 scores ranged from 1‐16 (mean = 8, *SD* 7). Three participants received a PHQ‐9 score that ranged from 0‐4 (minimal depression), one participant received a PHQ‐9 score in the range of 5–9 (mild depression), and one participant received a PHQ‐9 score in the range of 15–19 (moderately severe depression).

At 12 weeks' postdischarge follow‐up, the participants' PHQ‐9 scores ranged from 1‐12 (mean = 4, *SD* 4.6), three participants received PHQ‐9 scores in the range of 0–4 (minimal depression), one participant received a PHQ‐9 score in the range of 5–9 (mild depression), and the other participant received a PHQ‐9 score in the range of 10–14 (moderate depression). From the initial screening to 12 weeks' postdischarge, all five participants showed clinical improvement in their PHQ‐9 scores.

### 5.2 Veterans RAND (Research and Development) 12‐item Health Survey

The VR‐12 questionnaire responses provided the PCS and MCS scores. From the initial screening to 8 weeks and 12 weeks postdischarge, there were improvements in the mean scores of both the PCS and MCS. The patient's PCS scores steadily improved from baseline (31.3 *SD* 7.5) to 8 weeks (32.5 *SD* 10.2) to 12 weeks (39.86 *SD* 10.0). Similarly, the MCS scores also steadily improved from baseline (26.18 ± *SD* 3.4) to 8 weeks (44.72 *SD* 9.6) to 12 weeks (48.18 *SD* 9.1).

### 5.3 The Stanford Diabetes Questionnaire

The Stanford Diabetes Questionnaire collected information on participants' general health, symptoms, daily activities, blood glucose testing, physical activity, confidence about doing things, diet and medications, and use of medical care. At the beginning of the study, participants rated their general health as fair or poor. By 12 weeks' postdischarge follow‐up, there was an improvement in their general health and participants rated their general health as either good or very good.

At the beginning of the study, participants reported that much of the time they were discouraged by their health problems, they were fearful about their future health some of the time and much of the time they worried about their health and were frustrated by their health problems. At 8 weeks postdischarge, the participants were worried about or discouraged by their health problems only some of the time. At 12‐week postdischarge, participants reported that at no time did they worry about or experience frustration with their health problems. Also at 12‐week postdischarge, participants reported a decrease in the severity of their fatigue. However, there was no improvement in symptoms such as increased thirst, dry mouth, decreased appetite, nausea or vomiting, abdominal pain, frequent nocturia, blood glucose >300, morning headaches, nightmares, night sweats, lightheadedness, shakiness or weakness, and passing out or fainting. Pain and shortness of breath also did not decrease.

During the initial screening, participants reported moderate health‐based interference in their normal activities. By the 12‐week postdischarge follow‐up, participants reported a reduction in health‐based interference in their normal social activities with family or friends, hobbies, recreational activities, and household chores.

Regarding diabetes care, all participants reported that they had blood glucose monitors and test strips at home. At the beginning of the study, participants reported checking blood glucose before administering insulin and if they felt their blood glucose was low. At the completion of the study, there were no changes in the frequency of blood glucose monitoring. Also, there were no differences in adherence to insulin and oral hypoglycaemic agents. Participants reported some stretching and walked as the types of physical activities in which they engaged. By 12 weeks postdischarge follow‐up, participants reported an increase in their time spent walking as part of their regular exercise regimen.

At the initial interview, the participants reported receiving preventive care such as eye examinations and foot care in the podiatry clinics in the past. The participants did not schedule eye examination or podiatry clinic visits during the study period.

### 5.4 Emergency department use and hospitalization

For the 6 months immediately before enrollment in the study, the average emergency department visit and hospitalization for the participants was 2.6 (*SD* 1.5) and 2.4 (*SD* 1.7) respectively. At the 8‐week follow‐up, one of the participants was admitted to an inpatient unit for a planned lower extremity amputation. Another participant was admitted for a planned adrenalectomy during the interval between the 8‐week and 12‐week follow‐up interviews. During the study period, participants did not report any emergency department visits or unplanned hospitalizations.

### 5.5 Feasibility of depression screening

The researcher approached 30 patients and explained the study protocol. Of the 30 patients, 21 patients (70%) signed the consent and completed the depression screening. Seven patients screened positive for depression and enrolled in the study. Two patients were lost to follow‐up due to death. At the completion of the study, participants answered the survey that explored their experience with depression screening. All five participants reported feeling that they needed help regarding their depression and all five participants received medications for their depression. Three out of the five participants reported that, before the study, they knew about their depression, however, did not report their symptoms to their providers. None of the participants reported having any concerns about the medical provider discussing their depression or prescribing treatment for their depression. Of the five participants, four preferred that their medical provider manage their depression, whereas one participant had equal preference for either a medical or mental health provider. Finally, none of the participants had any suggestions for improving the care of their diabetes or depression.

At the conclusion of the study, of the 13 hospitalist providers, nine answered the anonymous survey regarding their experience related to the study protocol. All nine providers (100%) reported that they consider patients with diabetes as having a higher risk for developing depression. One provider had not considered this increased risk in the past; however, with this study and the provider learning session associated with the study, the provider now agrees that patients with diabetes have an increased risk of developing depression.

Of the nine providers who responded to the survey, 100% believed that depression screening and management needed to be integrated into the management of patients with diabetes. Also, the providers rated themselves as fairly confident in initiating depression management. Of the nine providers, three (33.3%) suggested that involving the mental health specialist through an electronic consult may improve the process of depression management for hospitalized patients. To initiate timely management of depression, providers suggested adding PHQ‐2 in the standard admission screening and providing routine depression screening during outpatient visits. Providers also reported that the information received in the provider education session conducted before the study and the handouts/pocket cards distributed to the providers were useful.

## 6 DISCUSSION

The purpose of this study was to explore the feasibility of depression screening and initiation of management for depression in patients with diabetes while they are hospitalized with a medical illness and to evaluate the effect of depression management on their depression, health‐related quality of life, diabetes self‐management, and healthcare use. The results of this study support the feasibility of screening for depression in hospitalized patients with diabetes and improvement in their depression, health‐related quality of life and diabetes self‐management as a result of managing their depressive symptoms. Also, the management of depression in patients with diabetes may decrease their emergency department use for nonemergent conditions and rehospitalizations for ambulatory care‐sensitive conditions.

### 6.1 Patient Health Questionnaire

Although the sample size in this study was too small to make a comparison, the improvement in depression in this study was similar to the improvement reported in one study in patients with diabetes who received medication management for their depression (Nicolau, Rivera, Francés, Chacártegui, & Masmiquel, 2013). Similarly, a Cochrane Review of psychological and pharmacological interventions for depression in patients with diabetes reported a clinically significant improvement in depression (Baumeister, Hutter, & Bengel, 2012). In the current study, the improvement in depression showed a noticeable trend over the 12‐week study period. Antidepressants require time and sometimes dosage adjustments to reach their full therapeutic effect. During this period, patients may continue to experience depression. These findings endorse the importance of enhanced support from the clinician and healthcare team to ensure treatment adherence in patients with diabetes and depression.

### 6.2 Veterans RAND (Research and Development) 12‐item Health Survey

At baseline, the participants' PCS and MCS scores from the VR‐12 were lower than the average scores of the general population. It is unclear whether the patient's status as a veteran was influencing their health‐related quality of life. Also, these lower PCS and MCS scores might reflect the poor physical health of the participants, especially when they were hospitalized for a medical illness. However, their poor quality of life could also be due to depression, as there is a reciprocal relationship between severity of depression and health‐related quality of life in patients with diabetes (Timar et al., 2016). Similar to reports from past studies (Filipcić, Margetić, Simunović, & Jakovljević, 2010; Nicolau et al., 2013), this study revealed improvements in participants' PCS and MCS scores. Since the PCS and MCS scores showed improvement over the 12‐week period and not at the 8‐week postdischarge, the improvement may not be attributable entirely to the participant not being in the hospital at the time of data collection. When accounting for the relative improvement of MCS scores over PCS scores at study follow‐up assessments, it suggests that the depression treatment and not change in environment (discharge from the hospital) may have accounted for the improvement. Moreover, the improvement in MCS scores exceeded the improvement in PCS scores. Similarly, a recent Medical Expenditure Panel Survey analysis (Alenzi & Sambamoorthi, 2016) among patients with diabetes and depression also reported improvement in MCS scores, but not in PCS scores, when their depression was managed by antidepressants alone.

### 6.3 The Stanford Diabetes Questionnaire

In the current study, the perceived improvement in general health did not occur until the 12‐week postdischarge, which indicates that merely not being hospitalized did not improve the participants' perception of their general health. Participants reported an improvement in symptoms such as fatigue, pain, polydipsia, hyperglycaemia, and hypoglycaemia. However, participants reported no improvement in symptoms such as increased thirst, dry mouth, decreased appetite, nausea or vomiting, abdominal pain, frequent nocturia, blood glucose > 300, morning headaches, nightmares, night sweats, lightheadedness, shakiness or weakness, and passing out or fainting. Pain and shortness of breath also did not decrease. When the health‐related quality of life substudy of Action to Control Cardiovascular Risk in Diabetes (ACCORD) trial analysed the factors affecting diabetes symptoms, the researchers reported a significant positive association of current depression (PHQ‐9 score > 10) with the severity of diabetes symptoms (Sullivan et al., 2012). In the current study, at 12‐week follow‐up, irrespective of persisting symptoms, participants were neither worried about their future health nor frustrated by their current health problems. This positive approach to their health problems may be a reflection of their improved confidence in managing their diabetes (Ludman et al., 2013).

### 6.4 Emergency department use and hospitalization

One of the positive outcomes of this study was the decrease in participants' emergency department use for nonemergent conditions and rehospitalizations for ambulatory care‐sensitive conditions. Pederson and colleagues (Pederson, Warkentin, Majumdar, & McAlister, 2016) claimed that the presence of depression at discharge from medical units predicted the possibility of readmission or death. Significantly, the two participants who enrolled in this study and were lost to follow‐up due to death reported severe depression at initial screening.

### 6.5 Feasibility of depression screening

In the current study, 33.3% of the population presented with depression. As per the VA guidelines, the facility provides routine annual depression screening in primary care. One study reported a low sensitivity for annual screening in identifying depression among patients with coronary artery disease, which may be true for patients with diabetes as well (Shankman, Nadelson, McGowan, Sovari, & Vidovich, 2012). Another researcher recommended screening for depression in hospitalized patients with medical illnesses (IsHak et al., 2017).

In the current study, even though some of the participants recognized their depression, they did not report their symptoms to their medical providers. However, after receiving the depression screening as part of this study, these participants readily accepted treatment when it was offered by their medical provider. Also, the participants preferred that their medical provider managed their depression. It is possible that patients who are reluctant to approach mental health providers with depression may receive management for their symptoms by their medical provider if their provider recognizes their symptoms and offers management. However, 9 of 30 patients (30%) approached did not agree to depression screening. This reluctance may reflect their refusal to participate in the study by signing the consent, answering all screening questionnaires, and agreeing to follow‐up telephone calls rather than a true refusal to depression screening. All participants accepted treatment for depression recommended by their inpatient provider, which supports patients' acceptance of depression treatment initiated by their medical provider while they were hospitalized with a medical illness.

The healthcare providers who responded to the survey recognized the increased risk for depression in patients with diabetes and agreed with the benefits of depression screening during hospitalization. Providers were confident about initiating management for depression and, at the same time, acknowledged the benefits of collaboration with a mental health provider. One of the providers who responded to the survey contemplated the possibility of “false positive screens” among hospitalized patients due to physical symptoms. However, previous studies (Karamchandani et al., 2015; Wagner et al., 2017) supported the feasibility and acceptability of depression screening in hospitalized patients using different screening tools, including the PHQ‐9, the screening tool used in this study (Kroenke et al., 2001).

Outcomes of this study uphold the role of healthcare professionals in recognizing depression in hospitalized patients with diabetes. Any team members can administer a screening questionnaire for depression with the proper training (IsHak et al., 2017). Nurses, healthcare providers, clinical social workers, case managers, and other professionals directly involved in patient care need ongoing education on the increased risk of depression in patients with diabetes and the warning signs of depression.

### 6.6 Challenges encountered

Of the 30 patients approached to participate in the study, nine patients (30%) declined to participate in the study. Patients' reluctance may be attributed to reluctance in signing the informed consent and being contacted over the telephone for follow‐up after discharge from the hospital. However, patients' reluctance may also be due to the stigma associated with mental illness such as depression.

### 6.7 Limitations

Outcomes of the current study concur with the existing evidence of unidentified depression in patients with diabetes. However, the current study has several limitations, which include a very small sample size, a short duration of the study and the absence of a control group. Of the seven participants initially enrolled in the study, two participants died during the study period. Secondary to the short duration of the study, the effect of management of depression on glycaemic control was not measured. Also, the applicability of these study results to all people with diabetes is questionable given the specialized population of veterans involved in this study.

Despite these limitations, this study addressed the feasibility of depression screening in inpatient settings and the effect of depression treatment on depression, health‐related quality of life, diabetes self‐management, and healthcare use by adults with diabetes and depression. Next steps will include a randomized controlled pilot study to test the initial efficacy of depression screening in patients with diabetes hospitalized in the Veteran's Affairs Medical Center.

## 7 CONCLUSION

The study confirms the presence of depression in patients with diabetes and the feasibility of routine screening for depression in patients with diabetes hospitalized with a medical illness. The study also offers opportunities for improvement in healthcare delivery with an interprofessional approach. Any team member can administer the screening questionnaire for depression with proper training. Moreover, nurses, healthcare providers, clinical social workers, case managers, and other professionals from those disciplines directly involved in patient care need ongoing education on the increased risk of depression in patients with diabetes, as well as on the warning signs of depression. Also, healthcare providers would benefit from ongoing education on depression management and opportunities for collaboration with inpatient mental health providers. Improved communication between inpatient providers and outpatient providers may ensure appropriate postdischarge follow‐up. Finally, further research on the long‐term effects of depression management in patients with diabetes may suggest future directions for care.

## ETHICAL STATEMENT

Human subjects' approval was granted by the Durham Veterans Affairs Medical Center, Durham, North Carolina, United States and the University of North Carolina at Chapel Hill, Chapel Hill, North Carolina, United States. All participants gave informed consent before involvement in the study.

## CONFLICT OF INTEREST

No conflict of interest has been declared by the authors.

## AUTHOR CONTRIBUTIONS

All authors have agreed on the final version and meet at least one of the following criteria [recommended by the ICMJE (https://www.icmje.org/recommendations/ )]:

- substantial contributions to conception and design, acquisition of data or analysis and interpretation of data;
- drafting the article or revising it critically for important intellectual content.
